# The role of bile acids in cellular invasiveness of gastric cancer

**DOI:** 10.1186/s12935-018-0569-0

**Published:** 2018-05-21

**Authors:** Yu-Chung Wu, Chang-Fang Chiu, Chung-Tzu Hsueh, Chung-Tsen Hsueh

**Affiliations:** 1 Taipei Veterans General Hospital and National Yang Ming University, No. 201, Section 2, Shipai Road, Beitou District, Taipei, 112 Taiwan; 2 China Medical University Hospital, Taichung, 404 Taiwan; 3 Department of Dentistry, Cathay General Hospital, Taipei, 106 Taiwan; 4 Division of Medical Oncology and Hematology, Department of Medicine, Loma Linda University, 11175 Campus Street, CSP 11015, Loma Linda, CA 92354 USA

**Keywords:** Bile acid, Cyclooxygenase-2, Prostaglandin E2, Chenodeoxycholic acid, Ursodeoxycholic acid, Gastric cancer, Cancer cell invasion

## Abstract

**Background:**

Bile acids have been implicated in the development of digestive tract malignancy by epidemiological, clinical and animal studies. The growth and transformation signaling by most of the bile acids is thought to be related to the induced cyclooxygenase-2 (COX-2) expression and increased production of prostaglandin E2 (PGE2). The highly hydrophobic bile acids such as chenodeoxycholic acid (CD) and deoxycholic acid can promote carcinogenesis and stimulate the invasion of colon cancer cells. On the contrary, ursodeoxycholic acid (UDCA), a less hydrophobic stereoisomer of CD, inhibits proliferation and induces apoptosis in colon cancer cells. We examined the effects of bile acid on human gastric cancer cells MKN-74.

**Methods:**

Early-passage human gastric cancer MKN-74 cells were used for drug treatment, preparation of whole cell lysates, subcellular extracts and Western blot analysis. The levels of PGE2 released by the cells were measured by enzyme inummoassay to indicate COX-2 enzymatic activity. Cellular invasion assay was performed in Boyden chamber.

**Results:**

Exposure of CD led to activation of protein kinase C (PKC) alpha, increased COX-2 expression and increased PGE2 synthesis. The induced COX-2 protein expression could be detected within 4 h exposure of 200 μM CD, and it was dose- and time-dependent. PGE2 is the product of COX-2, and has been reported to cause tumor invasion and angiogenesis in animal study. Safingol (SAF), a PKC inhibitor, suppressed the COX-2 protein expression and PGE2 production by CD in MKN-74. Furthermore, UDCA suppressed PGE2 production by CD but did not affect COX-2 protein expression induced by CD. Using a Boyden chamber invasion assay, both SAF and UDCA impeded CD induced tumor invasiveness of MKN-74 by 30–50%.

**Conclusions:**

Our results indicate that signaling of hydrophobic bile acid such as CD in gastric cancer cells is through PKC activation and COX-2 induction, which leads to increased cellular invasion. By perturbing the bile acid pool, UDCA attenuates CD-induced PGE2 synthesis and tumor invasiveness.

## Background

Bile acids have been implicated in the development of digestive tract malignancy [1, 2]. Epidemiological studies have suggested that the concentration and composition of fecal bile acids, amphiphilic derivatives of cholesterol, are important determining factors in the etiology of colon cancer [3, 4]. The primary bile acids, cholic acid and chenodeoxycholic acid (CD), are synthesized in the liver and are excreted into the duodenum where they facilitate absorption of dietary lipids [5]. Most of these bile acids are reabsorbed in the intestine; however, a small quantity remains unabsorbed and passes into the colon where it is converted to secondary bile acids, deoxycholic acid (DCA) and lithocholic acid, by enteric bacteria [6]. The highly hydrophobic bile acids such as CD and DCA can promote carcinogenesis and stimulate the invasion of colon cancer cells. It has been postulated that bile acids with increased hydrophobicity such as CD and DCA have a greater capacity to pass through cell membrane and modulate signaling cascades for tumorigenesis in normal colonic cells [7, 8]. Qiao et al. have reported that DCA suppresses wild-type p53 by stimulating proteasome-mediated p53 protein degradation in HCT-116 colon cancer cells [9].

On the contrary, ursodeoxycholic acid (UDCA), a less hydrophobic stereoisomer of CD, is a naturally occurring bile acid found in small quantities in normal human bile, and is indicated clinically for gallbladder stone dissolution. Interestingly, UDCA inhibits proliferation and induces apoptosis in colon cancer cells [10–12]. Results from experimental animal studies and clinical observation in ulcerative colitis have implied UDCA has chemopreventive action for colonic carcinogenesis [13–17]. Moreover, UDCA inhibits proliferation and induce apoptosis in colon cancer cells [18, 19]. Herein, we have examined the effects of bile acid on human gastric cancer cells MKN-74.

## Methods

### Cell culture and drug treatment

Early-passage human gastric cancer MKN-74 cells were established and characterized as described previously [20, 21]. Human colon cancer cell line HT-29 was obtained from ATCC. Human gastric cancer cell line SK-GT5 was established and characterized as described previously [22, 23]. All the cultures were maintained in standard MEM media supplemented with 100 units/ml penicillin, 100 μg/ml streptomycin and 20% heat inactivated normal calf serum (Gibco) at 37 °C in a humidified atmosphere of 5% CO_2_. Cells were checked for mycoplasma contamination at least every 6 months and consistently tested negative. MTT ([3-(4,5-dimethylthiazole-2-yl)-2,5-diphenyl tetrazolium bromide]), 3-phorbol 12-myristate 13-acetate (PMA) and bisbenzimide trihydrochloride (Hoechst 33258) were purchased from Sigma. Safingol (SAF) was purchased from Aventi Polar Lipids. The cytotoxicity of each agent for 24-h exposure in MKN-74 cells was determined by MTT assay [24], and the concentration causing less than 20% of growth inhibition was used in this study. Twenty-four hours after passage, when cells were approximately 50–60% confluent, they were exposed to the indicated drug treatment. Cells were checked for Mycoplasma contamination at least every 6 months with a GEN-Probe Mycoplasma rapid detection kit (Fischer Scientific) and consistently tested negative.

### Preparation of whole cell lysates and Western blot analysis

After drug treatment, cells were washed twice with cold phosphate-buffered saline, and then lysed by scrapping into a radioimmunoprecipitation assay buffer (phosphate-buffered saline containing 1% Nonidet P-40, 0.5% sodium deoxycholate and 0.1% SDS) containing the protease inhibitors (100 μg/ml phenylmethylsulfonyl fluoride, 25 μg/ml aprotinin, 25 μg/ml leupeptin, 10 μg/ml soybean trypsin inhibitor and 1 mM sodium orthovanadate). The lysate was left on ice for 30 min, passed through a 21-gauge needle twice, and then centrifuged at 15,000×*g* for 20 min in a microfuge at 4 °C. The clarified supernatant was collected and the protein concentration was measured using Bio-Rad protein assay kit (Bio-Rad, Hercules, CA). Sodium dodecyl sulfate–polyacrylamide gel electrophoresis (SDS-PAGE) was performed as previously described [25]. Fifty microgram of protein from each sample was run in SDS-PAGE using a Bio-Rad Mini-Protean system with an 8% resolving gel and 4% stacking gel. The resolved proteins were transferred onto Immobilon polyvinyl difluoride membranes (Millipore Corporation, Bedford, MA). Ponceau S (Sigma Chemical, St. Louis, MO) staining of the membranes was performed to assess the equivalence of sample loading and gel transfer. Antibodies purchased from Santa Cruz Biotechnology were used to detect the proteins of interest. After incubation with secondary antibody, membranes were developed using the Pierce SuperSignal chemiluminescent detection reagents (Pierce Biotechnology, Rockford, IL) according to the manufacturer’s instructions and exposed to NEN Renaissance X-ray film (New England Nuclear, Boston, MA) with intensifying screens. The linear-range signal intensity of each specific band on the fluorogram was quantitated by a densitometric scanning system and comparison of proteins of interest was performed after normalization to the densitometric scanning of the Ponceau S staining.

### Preparation of subcellular extracts

Membrane and cytosolic protein extracts were prepared based upon a method described previously [26]. Briefly, 2–4 × 10^6^ MKN-74 cells were suspended in ice-cold lysis buffer (20 mM Tris–HCl, pH 7.5, 2 mM EDTA, 2 mM [ethylenebis(oxyethylenenitrilo)]tetraacetic acid, 10 mM β-mercaptoethanol, 0.5 mM phenylmethylsulfonyl fluoride, 10 μg/ml leupeptin, 10 μg/ml soybean trypsin inhibitor and 10 μg/ml Aprotinin) at a concentration of 10^7^ cells/ml. Cells were lysed by passing through gauge 27 needle three times, and further homogenized by a motorized pestle for 50 strokes. The extent of cell lysis was examined under microscope throughout the homogenization, and consistent results with more than 95% cell lysis were obtained. Cell homogenates were centrifuged at 27,000×*g* for 30 min at 4 °C. The supernatants were collected as cytosolic protein extracts. The pellets were resuspended in ice-cold lysis buffer plus 1% Triton-X for 30 min at 4 °C, and intermittently homogenized by a motorized pestle every 10 min. The undissolved debris was removed after centrifugation at 12,000×*g* for 10 min, and the clarified supernatants were collected as membrane protein extracts. Protein concentration was determined by Bio-Rad protein assay. For Western blot analysis, 30 μg of protein from each extract was used.

### Measurement of prostaglandin E2 (PGE2) production

Cyclooxygenase-2 (COX-2) enzymatic activity was estimated by the production of PGE2 [27, 28]. Cells (1 × 10^4^/well) were plated in 24-well dishes and grown to 60% confluence in DMEM containing 10% FCS. The medium was then replaced with DMEM containing 1% FCS and vehicle or drug to be tested for 12 h. At the end of the treatment period, the culture medium was replaced with fresh DMEM containing 1% FCS and 1 μM sodium arachidonate. After 30 min, the medium was collected for analysis of PGE2. The levels of PGE2 released by the cells were measured by enzyme inummoassay using an ELISA kit from Oxford Biomedical Research. Rates of production of PGE 2 were normalized to protein concentrations.

### Invasion assay

The invasion assay was performed based on published methods with some modifications [29, 30]. Exponentially growing cells after 2–3 days of growth in complete medium were collected, and the old medium was saved as conditioned medium for chemotaxis. The Nuclepore filter was coated with 1 ml matrigel on ice, then incubated at 37 °C for 15–30 min. Boyden chamber was assembled with 24 μl of conditioned medium in each lower chamber, matrigel-coated Nuclepore filter facing upward, and 50 μl of serum-reduced (5% or less) medium containing 2.5 × 10^5^ cells in each upper chamber. After incubating the Boyden chamber in tissue culture incubator for 6 h, the filter was reversed, fixed in methanol, and stained by H&E. The cells that have traveled past the filter were counted as invasive cells.

### Statistical analysis

All experiments have been performed at least twice with similar results, and the results of one representative experiment are reported. Invasion assay results are reported as the average of three experiments with error bars representing standard error of the mean as shown in Fig. 4.

## Results

**Fig. 1:**
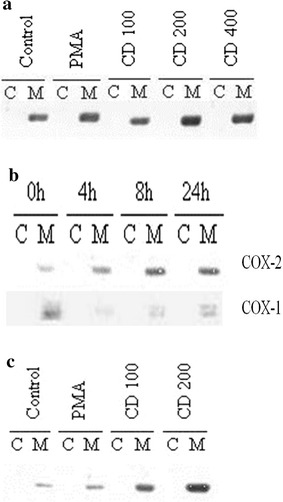
**a** Western blot analysis of COX-2 in MKN-74 after treatment with 10 nM PMA, 100, 200 and 400 μM CD, respectively for 24 h. M denotes membrane extract and C denotes cytosolic extract. **b** Western blot analysis of COX-2 and COX-1 in MKN-74 after treatment with 200 μM CD for 0, 4, 8 and 24 h, respectively. **c** Western blot analysis of COX-2 in SK-GT5 after treatment with 10 nM PMA, 100 and 200 μM CD, respectively for 24 h

We first examined the effect of CD in MKN-74 cells with regard to COX-2 expression. MKN-74 cells were exposed to 100, 200 or 400 μM CD for 24 h, then cells were collected and fractionated into membrane (M) and cytosolic (C) extracts as described in Methods. Western blots analyses were performed using COX-2 antibody. As shown in Fig. 1a, with exposure to 10 nM PKC activator PMA as a positive control, a dose-dependent induction of COX-2 protein expression by CD was demonstrated; similar results were noted in primary gastric adenocarcinoma SK-GT5 cells (Fig. 1c). Furthermore, a time-course induction of COX-2 without affecting COX-1 was seen after exposing to 200 μM CD at different time interval for MKN-74 cells (Fig. 1b). Similar results were noted in human colon cancer cell line HT-29 (data not shown).

**Fig. 2:**
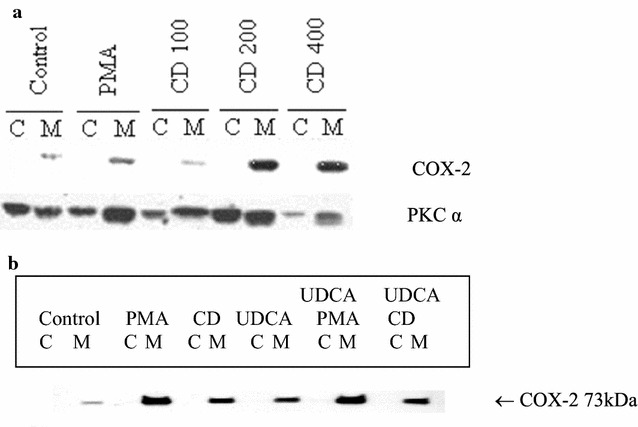
**a** Induction of cyclooxygenase (COX)-2 by CD required activation of PKCα in MKN-74 cells. Exponentially growing MKN-74 cells were exposed to control, 10 nM PMA, and CD in 100, 200 and 400 μM respectively for 24 h; then cells were collected and fractionated into membrane (M) and cytosolic (C) extracts. Western blots analyses were performed using COX-2 and PKCα antibody. Induction of COX-2 protein expression was associated with activation of PKCα, as demonstrated by translocation from cytosolic fraction to membrane fraction. **b** UDCA did not affect PMA- or CD-induced COX-2 protein expression. Exponentially growing MKN-74 cells were exposed to control, 10 nM PMA, 200 μM CD, 200 μM UDCA, 10 nM PMA plus 200 μM UDCA, or 200 μM CD plus 200 μM UDCA for 8 h, then cells were collected and fractionated into membrane (M) and cytosolic (C) extracts. Western blots analyses were performed using COX-2 antibody. UDCA did not affect PMA- or CD-induced COX-2 protein expression

We then examined the effect of CD on PKCα activation. MKN-74 cells were exposed to 100, 200 or 400 μM CD for 24 h; then cells were collected and fractionated as described. Western blots analyses were performed using COX-2 and PKCα antibody. As shown in Fig. 2a, induction of COX-2 protein expression was associated with activation of PKCα, as demonstrated by translocation from cytosolic fraction to membrane fraction.

We further examined the effect of UDCA on COX-2 expression. MKN-74 cells were exposed 8 h to single drug alone including PMA, CD and UDCA, 10 nM PMA with 200 μM UDCA, or 200 μM CD with 200 μM UDCA; then cells were collected and fractionated as described. Western blots analyses were performed using COX-2 antibody. As shown in Fig. 2b, UDCA did not affect PMA- or CD-induced COX-2 protein expression.

**Fig. 3:**
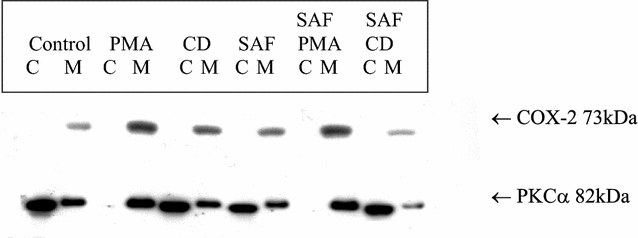
PKC inhibitor SAF suppressed CD-induced PKCα activation and COX-2. Exponentially growing MKN-74 cells were exposed to control, 10 nM PMA, 200 μM CD, 10 nM SAF, 10 nM PMA plus 10 nM SAF, or 200 μM CD plus 10 nM SAF for 8 h, then cells were collected and fractionated into membrane (M) and cytosolic (C) extracts. Western blots analyses were performed using PKCα and COX-2 antibodies

We then examined the effect of PKC inhibitor SAF on CD-induced PKCα activation and COX-2 protein expression. Exponentially growing MKN-74 cells were exposed 8 h to single drug alone including PMA, CD and SAF, 10 nM PMA with 10 nM SAF, or 200 μM UDCA with 10 nM SAF; then cells were collected and fractionated as described. As shown in Fig. 3, SAF suppressed CD-induced PKCα activation and COX-2 protein expression. There was no effect of low concentration of SAF (10 nM) on the induction of COX-2 and activation of PKCα from PMA; we previously showed higher concentration of SAF (50 nM) was needed to suppress PMA-induced PKCα activation and COX-2 protein expression [26].

**Fig. 4:**
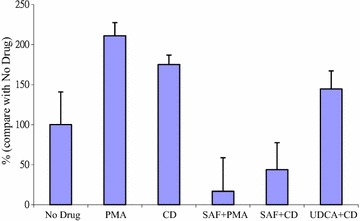
PKC inhibitor SAF and UDCA inhibited invasiveness in CD-exposed MKN-74. By using Boyden chamber to determine invasiveness in MKN-74 cells exposing to various agents as in Fig. 3, both SAF and UDCA blocked CD-induced invasiveness in MKN-74 cells. Values were reported as the average of three experiments with error bars showing standard error of the mean

We examined PGE2 production under various conditions; MKN-74 cells were exposed to single drug alone, 10 nM PMA with 200 μM UDCA, or 200 μM CD with 200 μM UDCA for 8 h, then PGE2 production was measured by ELISA. UDCA suppressed PGE2 production by PMA and CD (Table 1). Boyden chamber assay was performed to determine invasiveness in MKN-74 cells exposing to various agents. Either SAF or UDCA attenuated CD-induced invasiveness in MKN-74 cells (Fig. 4).

**Table 1:** PGE2 production determined by ELISA

Treatment group	PGE2 production (pg/μg total cellular protein)
Untreated	2.04
PMA	53.23
CD	3.37
UDCA	6.15
UDCA + PMA	22.28
UDCA + CD	2.05

UDCA suppresses PMA- and CD-induced PGE2 production. Exponentially growing MKN-74 cells were exposed to single drug alone, 10 nM PMA with 200 μM UDCA, or 200 μM CD with 200 μM UDCA for 8 h, then PGE2 production was measured by ELISA

## Discussion

Our data have shown that CD exposure in human gastric cancer MKN-74 cells leads to activation of PKCα and induction of COX-2 expression in conjunction with increased PGE2 production. Furthermore, UDCA suppresses CD-induced PGE2 production without affecting COX-2 expression. This implies that signaling of hydrophobic bile acid in gastric cancer is mediated through PKC activation and COX-2 induction, which gives rise to increased cellular invasion. By perturbing the bile acid pool, UDCA attenuates CD-induced PGE2 synthesis and tumor invasiveness without affecting the COX-2 expression.

We have used another cell line, HT-29 colon cancer cells as a control, for the induction of COX-2 protein expression by CD. CD has been shown to increase COX-2 protein expression via transcriptional regulation possibly through protein kinase C mediated mechanism in human colon cancer cells including HT-29 [31]. Pyo et al. have shown DCA inhibited tumor invasion and migration in gastric cancer cell lines, SNU-216 and MKN-45 [32]. It is worth noting that both MKN-74 and MKN-45 were derived from the metastatic liver tumors and SNU-216 was derived from lymph node metastasis [33, 34]. Moreover, we observed similar findings in primary gastric adenocarcinoma SK-GT5 cells. This indicates the effects of bile acid on gastric cancer invasion and migration are not cell line dependent.

Bile reflux has been implicated in the genesis of gastric and esophageal cancer in animals and humans [35–38]. Animal study in rats indicates that reflux of duodenal contents into the esophagus leads to increased COX-2 expression and mucosal thickening, and bile acids are likely to contribute to these effects [39]. Pulses of acid or bile have been shown to increase cell proliferation and COX-2 expression in Barrett’s esophagus epithelial cells [40]. Exposure of the CD and DCA in SK-GT4, a human esophageal cancer cell line, leads to a significant induction of COX-2 gene expression and a tenfold increase in the production of PGE2 [41].

It has been postulated that bile acids with increased hydrophobicity such as CD and DCA have a greater capacity to pass through cell membrane and modulate signaling cascades for tumorigenesis in normal colonic cells [7]. Qiao et al. have reported that DCA suppresses wild-type p53 by stimulating proteasome-mediated p53 protein degradation in HCT-116 colon cancer cells [42]. UDCA, a less hydrophobic stereoisomer of CD, is a naturally occurring bile acid found in small quantities in normal human bile, and is used clinically for gallbladder stone dissolution. Interestingly, UDCA is found to have anti-proliferative effects on normal intestinal cells [12]. Results from experimental animal studies and clinical observation in ulcerative colitis have pointed out that UDCA is a chemopreventive agent for colonic carcinogenesis [15]. Moreover, UDCA inhibits proliferation and induce apoptosis in colon cancer cells [10]. Most of the bile acids except UDCA stimulate the proliferation and invasion of human colorectal cancers [6].

Debruyne et al. have demonstrated that bile acids stimulate cellular invasion of human colorectal cancer cells at different stages of tumor progression [43]. Bile acid-stimulated invasion occurs through stimulation of haptotaxis and was dependent on the RhoA/Rho-kinase pathway and signaling cascades using PKC, mitogen-activated protein kinase, and COX-2. Hydrophobic bile acids such as DCA and CD have been shown to induce COX-2 gene expression through activation of PKC signal transduction in colorectal and esophageal cancer cells. Inhibitors of PKC can block the induction of COX-2 by DCA and CD [41, 44]. Bile acid-mediated induction of COX-2 can be important for tumorigenesis in the gastrointestinal tract because the products of COX-2 activity, e.g. PGE2, inhibit apoptosis, and increase the invasiveness and angiogenesis of malignant cells. Jacoby et al. reported a study using APC-mutant Min mouse model for FAP found that UDCA treatment decreased tumors throughout the entire intestine in a dose-dependent fashion, compared with control treatment [45]. Combined treatment with UDCA plus sulindac, an inhibitor of COX-1 and COX-2 that is active in the treatment of FAP, was more effective than either compound alone in reducing the incidence of intestine neoplasia.

Clinical studies have indicated that UDCA treatment was associated with decreased recurrence rates of colorectal adenomas in patients with a history of primary biliary cirrhosis and was associated with a lower prevalence of colonic cancer in patients with ulcerative colitis and primary sclerosing cholangitis [46, 47]. Alberts et al. reported a phase III doubled-blind placebo-controlled study involving 1285 adults who had undergone removal of a colorectal adenoma within the past 6 months to daily treatment with UDCA or placebo for 3 years [48]. UDCA treatment was associated with a non-statistically significant reduction in total colorectal adenoma recurrence but with a statistically significant 39% reduction in recurrence of adenomas with high-grade dysplasia.

## Conclusions

Bile acid has been implicated in the development of digestive tract malignancy such as colonic, gastric and esophageal neoplasms by epidemiological, clinical and animal studies. In colon cancer, the highly hydrophobic bile acids such as CD promote carcinogenesis and stimulate the invasion; UDCA, a less hydrophobic stereoisomer of CD, inhibits proliferation and induces apoptosis. Our investigation in gastric cancer indicates signaling of CD is mediated by activation of PKC and COX-2, which leads to increased cellular invasion. Either UDCA or PKC inhibitor can interfere signaling of CD, causing decreased cellular invasion.

## Abbreviations

CD: chenodeoxycholic acid
DCA: deoxycholic acid
UDCA: ursodeoxycholic acid
MTT: 3-(4,5-dimethylthiazole-2-yl)-2,5-diphenyl tetrazolium bromide
PMA: 3-phorbol 12-myristate 13-acetate
SAF: Safingol
PKC: protein kinase C
PGE2: prostaglandin E2
COX-2: cyclooxygenase-2
M: membrane extract
C: cytosolic extract
